# Head Exposure to Cold during Whole-Body Cryostimulation: Influence on Thermal Response and Autonomic Modulation

**DOI:** 10.1371/journal.pone.0124776

**Published:** 2015-04-27

**Authors:** Julien Louis, Karine Schaal, François Bieuzen, Yann Le Meur, Jean-Robert Filliard, Marielle Volondat, Jeanick Brisswalter, Christophe Hausswirth

**Affiliations:** 1 Research Department, Sport Expertise and Performance (SEP) Laboratory, French National Institute of Sport, Expertise and Performance (INSEP), Paris, France; 2 Sports Performance Laboratory, Sports Medicine Program, University of California Davis, Sacramento, California, United States of America; 3 Medical Department, French National Institute of Sport, Expertise and Performance (INSEP), Paris, France; 4 Laboratory of Human Motricity, Education Sport and Health, University of Nice Sophia Antipolis, Nice, France; The Ohio State University, UNITED STATES

## Abstract

Recent research on whole-body cryotherapy has hypothesized a major responsibility of head cooling in the physiological changes classically reported after a cryostimulation session. The aim of this experiment was to verify this hypothesis by studying the influence of exposing the head to cold during whole-body cryostimulation sessions, on the thermal response and the autonomic nervous system (ANS). Over five consecutive days, two groups of 10 participants performed one whole-body cryostimulation session daily, in one of two different systems; one exposing the whole-body to cold (whole-body cryostimulation, WBC), and the other exposing the whole-body except the head (partial-body cryostimulation, PBC).10 participants constituted a control group (CON) not receiving any cryostimulation. In order to isolate the head-cooling effect on recorded variables, it was ensured that the WBC and PBC systems induced the same decrease in skin temperature for all body regions (mean decrease over the 5 exposures: -8.6°C±1.3°C and -8.3±0.7°C for WBC and PBC, respectively), which persisted up to 20-min after the sessions (P20). The WBC sessions caused an *almost certain* decrease in tympanic temperature from Pre to P20 (-0.28 ±0.11°C), while it only decreased at P20 (-0.14±0.05°C) after PBC sessions. Heart rate *almost certainly* decreased after PBC (-8.6%) and WBC (-12.3%) sessions. Resting vagal-related heart rate variability indices (the root-mean square difference of successive normal *R-R* intervals, RMSSD, and high frequency band, HF) were *very likely* to *almost certainly* increased after PBC (RMSSD:+49.1%, HF: +123.3%) and WBC (RMSSD: +38.8%, HF:+70.3%). Plasma norepinephrine concentration was *likely* increased in similar proportions after PBC and WBC, but only after the first session. Both cryostimulation techniques stimulated the ANS with a predominance of parasympathetic tone activation from the first to the fifth session and in slightly greater proportion with WBC than PBC. The main result of this study indicates that the head exposure to cold during whole-body cryostimulation may not be the main factor responsible for the effects of cryostimulation on the ANS.

## Introduction

Over the last decade, there has been increasing interest in the study of the influence of the autonomic nervous system (ANS) on the modulation of cardiac activity. Specifically, the link between a diminished parasympathetic modulation of heart rate and the occurrence of cardiovascular diseases or altered post-exercise recovery has been studied as well in the medical domain as in the sporting realm [1]. At rest, the equilibrium between the two branches of the ANS confers a cardioprotective background; the sympathetic branch acting as an accelerator of heart rate through humoral and neural pathways, and the parasympathetic branch acting as a brake on heart rate through neural pathways. Strong positive correlations have been reported between the magnitude of cardiodeceleration after exercise and health [2,3]. At the opposite, impairment or postponement of parasympathetic activation after exercise has been shown to be associated with an increased risk of cardiovascular accident such as sudden cardiac death. Some studies suggest that myocardial exposure to high levels of norepinephrine may result in β receptor-mediated cytotoxic effects and apoptosis as well as α receptor-mediated hypertrophic effects [4,5], dramatically increasing the risk of mortality [1]. The prevention of such effects by increasing vagal activity has become a priority for researchers.

Body exposure to cold is well known to alter the modulation of the ANS. Cold stimulation triggers peripheral vasoconstriction, leading to a shift in blood volume toward the core [6,7]. The resulting increase in central pressure in turn activates the baroreflex, responsible for reducing sympathetic nerve activity while shifting autonomic heart rate control toward a parasympathetic dominance. Numerous studies have shown that water immersion is an effective strategy to increase parasympathetic activity, particularly after exercise in athletes [8–10]. More generally, cooling the body after exercise has become a natural recovery strategy commonly used in numerous sports. Body exposure to cold has been shown to aid recovery by altering blood flow [11] and improving perceptions of recovery [9,12]. Body exposure to cold may also exert important effects on post-exercise recovery at the cardiovascular level [13]. As exercise causes an intensity-dependent parasympathetic withdrawal and sympathetic increase, a prompt recovery of parasympathetic activity is desirable after exercise. For example, Stanley et al. [9] reported that both cold water immersion (CWI, 5-min in 14°C water) and contrast water therapy (CWT, alternating 1-min in 14.2°C and 2-min in 35.5°C water), significantly aided post-exercise parasympathetic reactivation compared to passive recovery in endurance trained athletes. They also reported that this effect was larger with CWI than CWT, suggesting that combining a greater cold stimulus increased the effectiveness of water immersion. Contrary to water immersion protocols, the effects of dry air whole-body cryostimulation (WBC, classically range from -110°C to -160°C) on post-exercise autonomic recovery is not well documented, even though this recovery method has become increasingly used in the sporting realm [12,14]. However, thanks to a very cold stimulus, WBC could induce larger parasympathetic activation than cold water immersion [15]. Accordingly, significant increases in heart rate variability (HRV) indices of parasympathetic activity (the root-mean square difference of successive normal R-R intervals, RMSSD, and high frequency band, HF) have been reported after a WBC session performed as well after exercise in elite synchronized swimmers [15] as in healthy nonathletic women [16]. More recently, in a study from our team, we also recorded a significant increase in parasympathetic activity within the 20-min following a single WBC session performed without previous exercise, as inferred through an increase in HF and RMSSD concomitant to a decrease in HR [13].

Interestingly, in the latter study by Hausswirth et al. [13] the increase in parasympathetic activity was significantly larger when participants performed their WBC session in a closed cryochamber exposing the whole body to a -110°C air, compared to a cryotherapy tank exposing the whole body, except the heat and neck, to a -160°C expanded nitrogen gas (called partial-body cryostimulation, PBC). According to this study, the effect of cold on the head alone may accentuate the parasympathetic activation. It has been shown that the direct effect of cold on the head alone, via face immersion in cold water (without breath holding) aided parasympathetic reactivation significantly following exercise [8,17]. This increase in vagal tone in response to cold stimuli applied to the face would be principally mediated by trigeminal brain stem pathways rather than by the baroreflex [18,19]. Superficial cold receptors that are innervated by the ophthalmic branch of the trigeminal nerve would enhance the cardiovagal activity [19–21]. However, in the last study from Hausswirth et al. [13], the larger increase in parasympathetic activity at the cardiac level obtained after WBC compared to PBC could be explained by both the head exposure to cold and the greater fall in skin temperature obtained after WBC, without being able to discern the relative influence of each.

Within this framework, the aim of this study was to examine the effect of two different strategies of cryotherapy (exposing or not exposing the head to cold) that induce the same decline in skin temperature in order to isolate the effect of head exposure to cold on thermal response and autonomic modulation. We hypothesized that exposing the head to the cold stimulus with the rest of the body would elicit a greater parasympathetic stimulation than cold exposure from the neck down and that the autonomic response would become attenuated with consecutive cryostimulation sessions. For each strategy, five cryostimulation sessions were performed over consecutive days, in order to determine whether any adaptive changes occurred in the magnitude of the autonomic response to cold.

## Materials and Methods

### Participants

Thirty healthy males volunteered to participate in this study (see Table 1 for characteristics). Before the experiment a physician examined all the participants to check they did not present contraindications to cold exposure such as cold hypersensitivity (Raynaud’s phenomenon), history of heart disease or circulatory pathologies. All participants were recreational athletes between 20 and 55 years of age and were not accustomed to cryotherapy treatments. Since the body fat mass can influence the physiological response to cryostimulation [22], body composition was controlled and measured using an 8 point bio-impedance device (InBody 720; 1–1000 kHz, Biospace company, Ltd., Seoul, Korea) validated for accuracy and repeatability [23,24]. All participants were requested not to smoke, and not to drink any alcohol or hot drinks for 4 h prior to each cold exposure in order to avoid influencing the recorded variable. In addition, participants were required not to undertake exercise for 24h prior to each laboratory session. All participants were volunteers and were informed about the study protocol, the risks of all tests, and their rights according to the Declaration of Helsinki (1964, revised in 2001). Participants gave their written informed consent and the study was approved by the local Ethics Committee (Ile-de-France VI, Paris, France) before its initiation.

**Table 1:** Characteristics of participants composing the three experimental groups (Control, WBC, and PBC).

	Control	WBC	PBC
N	10	10	10
Age (year)	33.9 ± 12.3	34.8 ± 9.1	34.7 ± 11.5
Height (m)	1.77 ± 0.06	1.74 ± 0.09	1.80 ± 0.1
Body mass (kg)	74.4 ± 11.8	69.9 ± 12.3	76.7 ± 8.6
BMI (kg.m^2^)	23.6 ± 3.1	23.0 ± 2.2	24.2 ± 3.0
Fat mass (%)	13.3 ± 6.0	14.2 ± 4.1	14.3 ±5.8

(Data are means ± SD).

BMI, Body Mass Index.

### Study design

**Fig 1:**
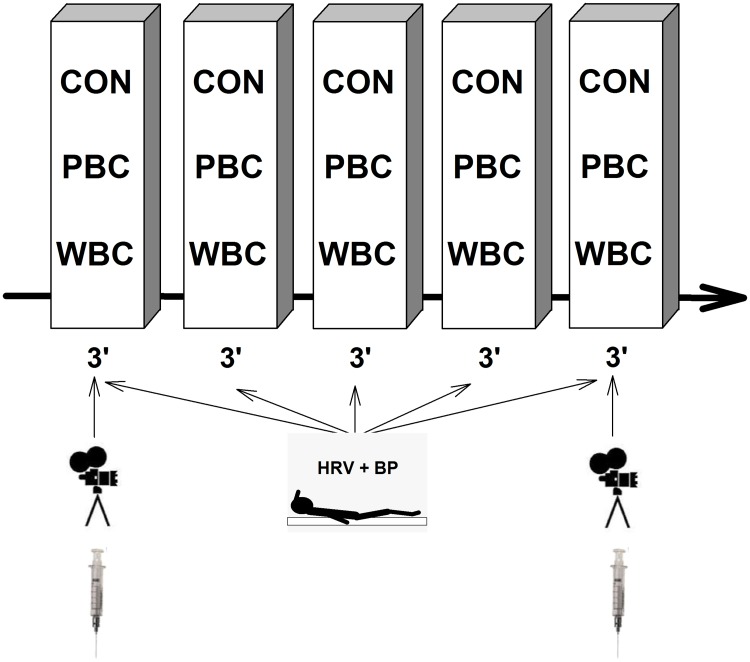
Schematic representation of the experimental protocol. Each subject experienced five consecutive (one session per day over five consecutive days) whole-body cryostimulation (WBC) or partial-body cryostimulation (PBC) sessions or no session (CON) for 3-min, immediately preceded and followed by the same measurements. Blood, and camera recordings were performed only on the first and fifth session. *HRV + BP*, measurements of heart rate variability and blood pressure.

This study was conducted to analyze the influence of head exposure to cold or not during repeated cryostimulation sessions on thermal and physiological variables. Two cryotherapy techniques were used; one system consisted of a cryochamber where the participants were entirely exposed to a very dry and cold air at -60°C (whole-body cryostimulation, WBC), whereas the other system was an open tank exposing the body to -160°C, except the head and neck (partial-body cryostimulation, PBC). In separate weeks, 3 groups of 10 participants were exposed either to five consecutive (one session per day) WBC or PBC sessions or to control sessions (24°C) for 3-min. The design of the study was a randomized controlled trial. The randomization procedure of the participants was administered by an assistant not involved in the experiment (i.e. draw from a hat). According to a protocol used in a previous study [13], physiological measurements were performed each day immediately before (Pre), immediately after (Post), every minute for the first 5 minutes post-exposure (P1 through P5) and 20-min after the exposure (P20) (Fig 1).

### Cryostimulation sessions

The investigations were carried out at the medical department of the National Institute of Sport, Exercise and Performance (INSEP, Paris, France), where two different cryotherapy systems were installed. One system consisted of three closed cryochambers displaying different temperatures; -10°C, -60°C and -110°C (Zimmer Elektromedizin, GmbH, Ulm, Germany), whereas the other system consisted of an open tank where all the body was exposed to a very cold temperature (-160°C) except the head and neck (Cryotechno, TEC4H, France). In both systems, the entire cooling process was automatically controlled and the temperatures of exposition remained constant throughout the experiment. The temperature provided by the Zimmer system was the mean ambient temperature recorded inside the cryochamber, whereas the temperature provided by the Cryotechno system was that recorded at the entrance of the tank, rather than the mean ambient temperature. In order to isolate the effect of head exposure to cold it was necessary to obtain a similar decline in skin temperature between the two cryotherapy systems. Accordingly, in preliminary testing sessions some additional participants were instructed to stay 3-min in the cryochamber of the Zimmer system either at -10°C, -60°C or -110°C, and in the open tank of the Cryotechno system. Finally, the -60°C cryochamber was chosen for the study since it matched the best with the body cooling obtained with the opened Cryotechno system. In the WBC modality, participants remained in the -60°C room for 3-min, while in the PBC modality they remained in the tank and received a very cold air at -160°C for 3-min. The main differences between the two systems were that whole body was exposed to a cold and dry air in the Zimmer system whereas expanded nitrogen gas was used in the Cryotechno system, and the head and neck were not exposed to cold. A familiarization session was previously organized with a time exposure reduced to 1-min, and the next cryostimulation sessions were administered under medical supervision. Moreover, before the cold exposure, the participants were instructed to towel-dry eventual sweat, wear a bathing suit, surgical mask, earband, triple layer gloves, dry socks and clogs. All jewelry, piercings, glasses and contact lenses were removed before the cold exposure. For the WBC, participants were instructed by the machine operator to walk slowly around the chamber and to flex and extend their elbows throughout the exposure. For the PBC, because of the small space inside the cryotherapy tank, participants could only slowly rotate and move their wrists slowly. An active surveillance of pre-defined adverse events relating to cryostimulation [25] was performed during the sessions and within a month after the study.

In addition, the air temperature and the relative humidity of the rooms used for measurements were recorded so that it was possible to control the confounding effects of potential changes in the environment.

### Measurements

#### Skin and tympanic temperatures

Skin temperature was assessed before and after the 1^st^ and the 5^th^ cryostimulation sessions, on Monday and Friday of the same week, respectively, by using a ThermoVision SC640 thermal imaging camera (Flir Systems, Danderyd, Sweden) in accordance with the standard protocol of infrared imaging in medicine [26]. The camera, with the emissivity set in the range of 0.97 to 0.98, was connected to a personal computer with appropriate software (Thermacam Researcher Pro 2.10, Flir systems, Danderyd, Sweden). The camera was mounted on a tripod and positioned in a way to focus on the entire body. The distance between the camera and the subject ranged from 2.5–3.0m (depending on the height and the size of the individual). The thermograms of the chosen body regions of interests were performed before (Pre) and during the first 5-min following the WBC and PBC sessions (Post to P5) and 20-min after (P20) in the temperate room where the temperature was maintained stable (24°C). Participants were instructed to remain standing in the anatomical position while the thermograms were performed. Participants were also asked to turn round immediately after the exposure to cold (Post), and at the end of each minute (P1 to P5) in order to perform thermograms of the back side. Finally, a last front and back thermogram was performed 20-min after the end (P20) of the WBC and PBC sessions. 22 body regions of interests were chosen to study as thoroughly as possible the evolution of skin temperature before and after the cold exposure. 12 regions corresponded to the anterior aspect of the body (i.e. right and left cheeks, torso, abdominal, right and left forearms, right and left arms, right and left thighs, and right and left legs) and 10 regions to the posterior aspect (i.e. upper back, lower back, right and left forearms, right and left arms, right and left thighs, and right and left legs) covering almost the whole body. For more clarity in the presentation of the results, the body regions corresponding to cheeks, the arms and forearms as well as for legs and thighs, and torso and abdominal, and upper back and lower back, were grouped, giving a mean skin temperature for the head, arms, legs, and trunk for the front and the back faces. The head temperature was that recorded on the right and left cheeks, more precisely the surface of the skin not covered by the mask or the earband in order not to be influenced by any piece of tissue on the skin. A mean temperature for the whole body surface was also calculated by averaging the skin temperature recorded for the 22 regions of interests.

Before and after each WBC and PBC session (from Monday to Friday of the same week), tympanic temperature (Ttymp) was also measured with a tympanic thermometer (Braun Thermoscan Pro 4000, NY, USA). This measurement was performed at Pre, Post, P5, and P20. For all measures of skin and tympanic temperature, the participants were standing up in front of the camera and worn only a bathing suit. Immediately after the cryostimulation session, they were instructed to take off as fast as possible their protective equipment and not to wear any personal protective equipment up to the last temperature recording in order not to influence the body warming.

#### Blood pressure, heart rate, and HRV indices of parasympathetic activity

Heart rate (HR) was recorded each day at Pre and P5. For each condition, participants were comfortably installed in a supine position on a medical bed for 8-min. This test was organized in a dark and quiet room, avoiding any distractions that could induce HR fluctuations. Additionally, the participants were asked to remain still and not to talk. For all resting HR recordings, *R-R* intervals were recorded continuously with a Suunto MemoryBelt HR monitor with a sampling rate of 1000 Hz, and the capacity to record respiratory rate (MemoryBelt, SuuntoOy, Vantaa, Finland).

*R–R* interval data files were transferred to the computer using the Suntoo Training Manager Software and were further analyzed using specialized heart rate variability (HRV) analysis software (Nevrokard aHRV, Izola, Slovenia). An experienced investigator visually identified and manually removed any occasional ectopic beats and artifacts. Since HRV parameters classically used to study the sympathetic modulation (i.e. SD2 and the low to high frequency ratio) are still a matter of debate, the HRV analysis was restricted to indicators of parasympathetic modulation. The time-varying index kept for analysis was the root-mean square difference of successive normal *R-R* intervals (RMSSD) [27]. Mean HR was also analyzed. Power spectral density analysis was then performed using a fast Fourier transform with a non-parametric algorithm. The power density of high frequency (HF: 0.15–0.50 Hz) component of the spectrum was calculated to provide an additional index of parasympathetic activity. Both HRV indices of parasympathetic activity were calculated using the last 4-min of the 8-min HR recordings [27]. Moreover, we decided to allow our participants to breathe spontaneously during the measurements [28]. For all HRV samples, it was verified that the respiration rate always remained in the high frequency range (HF: 0.15–0.50 Hz) since the system employed allowed to record this parameter during each test. When this assumption was not met, the test was not retained for subsequent analysis.

Systolic and diastolic blood pressures (Sys BP and Dia BP) were also recorded at the end of the 8-min resting period, by using an oscillometric sphygmomanometer (705 IT, Omron, Kyoto, Japan) positioned on the left arm while the person was still in a lying position.

#### Blood analyses

To avoid inter-assay variation, all blood samples were analyzed in a single batch at the end of the study. In two occasions (before and 25min after the 1^st^ and 5^th^ cryostimulation or control sessions), blood samples were collected from a superficial forearm vein using standard venipuncture techniques. 15 mL (2 tubes = 6mL and 1 tube = 3mL) of blood was directly collected into EDTA tubes for each sample (Greiner Bio-one; Frickenhausen, Germany).

Blood samples were immediately centrifuged at 3000 rev.min^-1^ for 10 min at +4°C to separate plasma from red blood cells. The obtained plasma sample was then stored in multiple aliquots (Eppendorf type, 1500 μL per sample) at -80°C until analysis. A sensitive high-pressure liquid chromatography (Knauer, Berlin, Germany; Column, Lichtopher 60, RP Select B, Merck, Germany) was used for further analysis. Plasma epinephrine and norepinephrine, were determined by means of an electrochemical detector (2143-RPE, Pharmacia LKB, Freiburg, Germany) and computed as ng.L^-1^.

### Statistical analysis

We tested the normality of each variable from a normal probability plot and by using the Shapiro-Wilk test. These analyses were performed using Statistica software (7.0 version, Statsoft, France). Because the cardiovascular and blood parameters data did not always meet the assumptions of normality, they were log-transformed to reduce bias arising from non-uniformity error. Data were then analyzed for practical significance using magnitude-based inferences [29]. All qualitative analyses were conducted using a modified statistical spreadsheet [30]. We used this qualitative approach because traditional statistical approaches often do not indicate the magnitude of an effect, which is typically more relevant for clinical or practical prescription than any significant effect. Further, considering the methodological factors that influence assessment of heart rate variability (likely contributing to its moderate level of reliability) [31], evaluation of meaningful changes in the cardiac parasympathetic response requires evaluation of the smallest worthwhile change [32]. The magnitude of the within-group changes or between-group differences in the changes, were interpreted by using values of 0.3, 0.9, 1.6, 2.5 and 4.0 of the within-athlete variation in the control group (coefficient of variation, CV in the control group) as thresholds for *small*, *moderate*, *large*, *very large* and *extremely large* differences in the change between the trials [33]. Quantitative chances of higher or lower differences were evaluated qualitatively as follows: <1%, *almost certainly not*; 1–5%, *very unlikely*; 5–25%, *unlikely*; 25–75%, *possible*; 75–95%, *likely*; 95–99%, *very likely*; >99%, *almost certain*. If the chance of higher or lower differences was >5%, the true difference was assessed as unclear. Otherwise, we interpreted that change as the observed chance [29]. Data in text and figures are presented as mean±90% confidence interval.

## Results

All data of all subjects were taken into account in the presentation of the results.

### Skin and tympanic temperatures

**Fig 2:**
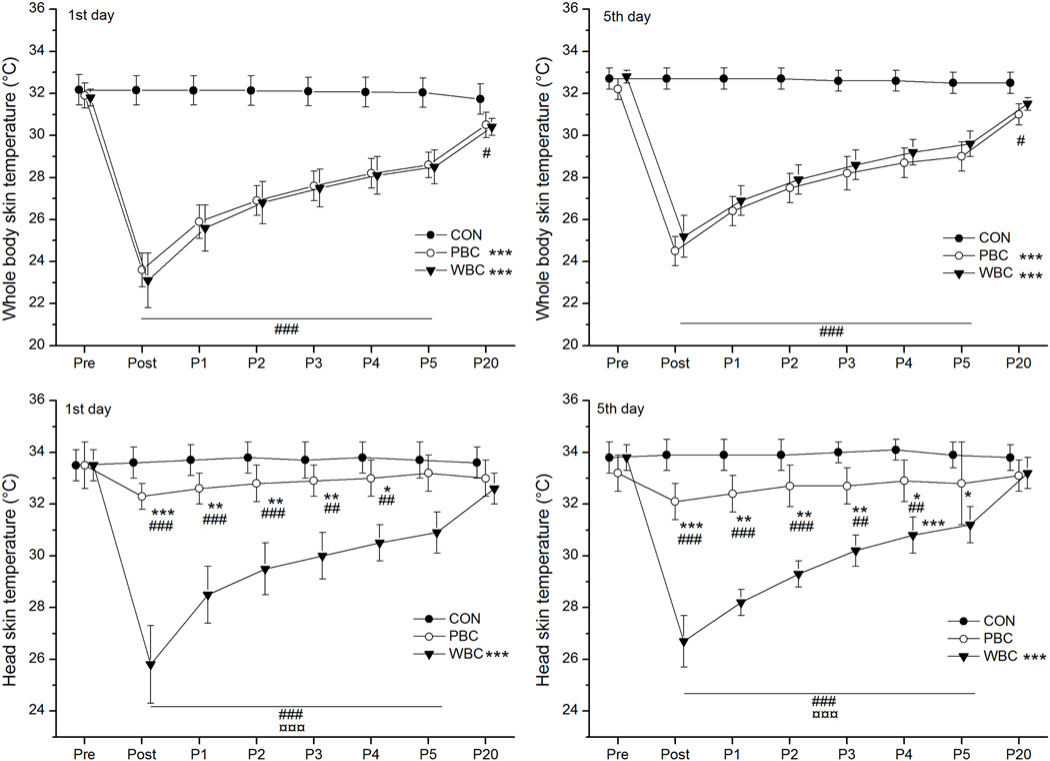
Changes in the mean skin temperature of the whole body (except the head) and the head alone. Values were recorded before (Pre), immediately after (Post) and for 20-min (P1 to P20) after the first and the fifth whole-body cryostimulation (WBC), and partial-body cryostimulation (PBC) sessions, and in the control (CON) condition. Within-group change (Post conditions vs. Pre): * likely; ** very likely; *** almost certain. Between-group (vs. CON) difference in the change: # likely; ## very likely; ### almost certain. Between-group (PBC vs. WBC) difference in the change: ¤ likely; ¤¤ very likely; ¤¤¤ almost certain.

Baseline skin temperature (Tskin) for all body regions was similar between groups before the cryostimulation, without difference between the first and fifth cryostimulation session (Fig 2). Each day, an *almost certain* very large reduction in mean skin temperature of the whole body was recorded within the 20-min after the PBC and WBC exposures (Fig 2). However, no difference was recorded between cryotherapy modalities from Post to P20. In both modalities, the mean Tskin *almost certainly* remained lower than baseline values up to P20 (Fig 2). In both modalities, a *very likely* large reduction in Tskin of the head was recorded in Post and persisted up to P5, in larger proportion for WBC than PBC (Fig 2). In both cryotherapy modalities, the mean decrease in Tskin for the whole body recorded immediately after (Post) the first cryostimulation session (-8.3±0.7°C and -8.6°C±1.3°C for PBC and WBC, respectively) was *very likely* larger than that recorded after the fifth cryostimulation session (-7.6±0.8°C and -7.7±0.9°C for PBC and WBC, respectively).

**Fig 3:**
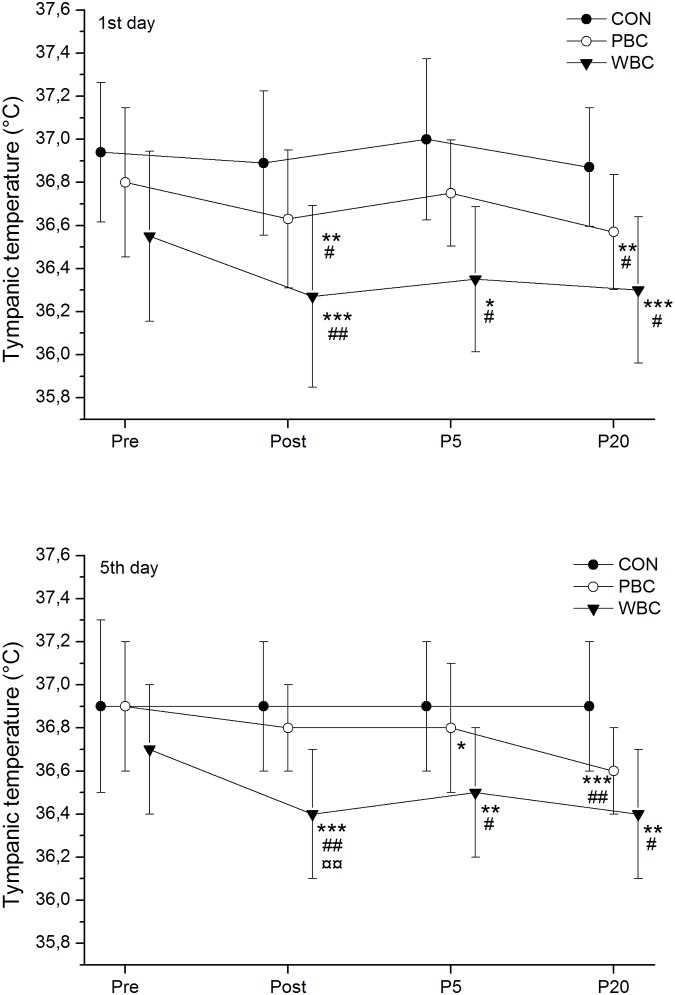
Changes in tympanic temperature. Values were recorded before (Pre), immediately (Post), 5-min (P5), and 20-min (P20) after the first and the fifth whole-body cryostimulation (WBC), and partial-body cryostimulation (PBC) sessions, and in the control (CON) condition. Within-group change (Post conditions vs. Pre): * likely; ** very likely; *** almost certain. Between-group (vs. CON) difference in the change: # likely; ## very likely; ### almost certain. Between-group (PBC vs. WBC) difference in the change: ¤ likely; ¤¤ very likely; ¤¤¤ almost certain.

The tympanic temperature (Ttymp) *almost certainly* decreased after the WBC sessions by -0.28±0.18°C on day 1 and -0.34±0.25°C on day 5 and *almost certainly* remained lower than baseline values up to P20 (Fig 3). There was a trivial difference in the decrease in Ttymp between the first and the last WBC sessions. A small to moderate reduction in Ttymp was recorded after PBC sessions, occurring *almost certainly* in P20 (-0.14±0.05°C). The reduction in Ttymp over the week was *very likely* larger after WBC than PBC in Post (-0.31±0.15°C vs. -0.07±0.12°C for WBC and PBC, respectively) and P5 (-0.26±0.15°C vs. -0.04±0.10°C for WBC and PBC, respectively).

### Cardiovascular parameters

**Fig 4:**
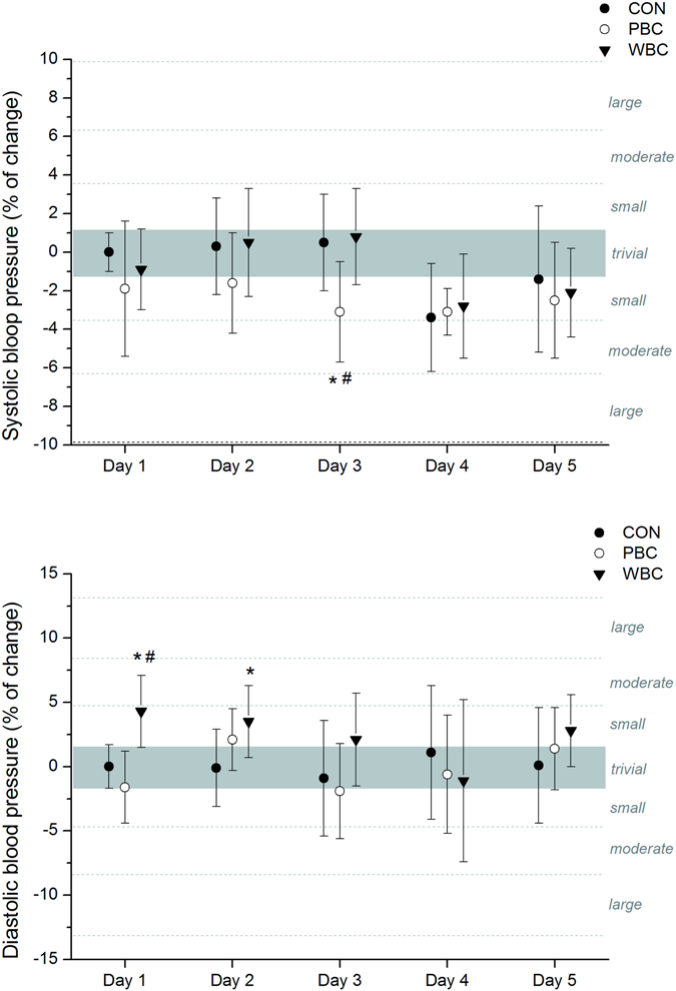
Changes (% of pre values) in systolic and diastolic blood pressures from pre to post whole-body cryostimulation (WBC), and partial-body cryostimulation (PBC) sessions, and in the control (CON) condition. The shaded area represents trivial changes. Values were recorded daily on five consecutive days before and after each cryostimulation sessions. Between-group (PBC or WBC vs. CON) difference in the change: * likely; ** very likely; *** almost certain. Between-group (PBC vs. WBC) difference in the change: # likely; ## very likely; ### almost certain.

No marked changes in systolic and diastolic BP were recorded after the cryostimulation sessions in PBC and WBC modalities. Only a *likely* small decrease in systolic BP was recorded after the third PBC session and a *likely* small increase in diastolic BP after the two first WBC sessions, but these increases disappeared for the next sessions (Fig 4).

**Fig 5:**
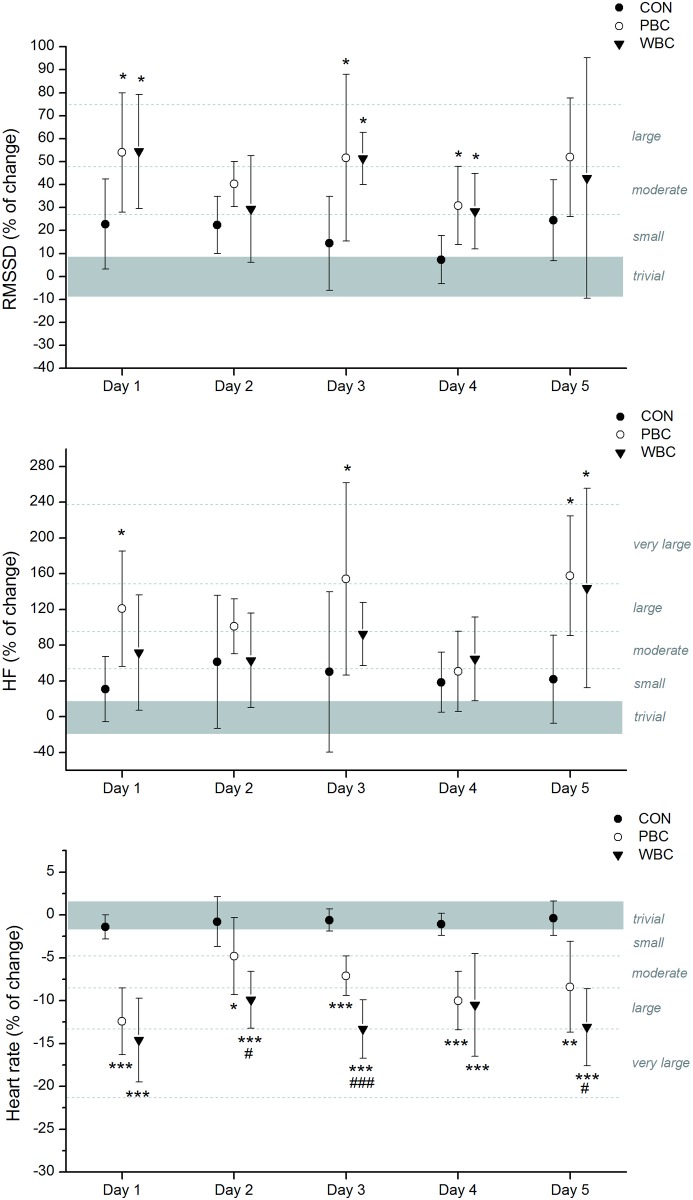
Changes (% of pre values) in heart rate and HRV indices of parasympathetic activity from pre to post whole-body cryostimulation (WBC), and partial-body cryostimulation (PBC) sessions, and in the control (CON) condition. The shaded area represents trivial changes. Values were recorded daily on five consecutive days before and after each cryostimulation session. Between-group (PBC and WBC vs. CON) difference in the change: * likely; ** very likely; *** almost certain. Between-group (PBC vs. WBC) difference in the change: # likely; ## very likely; ### almost certain.

In addition, HR *almost certainly* decreased in moderate to very large proportions after each PBC and WBC sessions, without significant differences over the week (Fig 5). The mean decrease obtained over the five cryostimulation sessions was -5.02±1.07 bpm after PBC and -7.7±2.3 bpm after WBC, and these changes were *almost certainly* different from the CON group. The decrease in HR was *likely* to *almost certainly* greater after WBC than PBC sessions in day 2, 3 and 5.

HRV indices of parasympathetic activity, namely RMSSD and HF were *likely* to *very likely* increased in moderate to large proportions after each WBC and PBC session, without marked differences between the modalities or between the first and the fifth session (Fig 5). The mean Pre-Post increases in RMSSD over the week were +49.1±15.4% and +38.8±14.7% in the PBC and WBC modalities, respectively, while HF increased by 122.3±40.0% and 70.3±29.5% in the PBC and WBC modalities, respectively.

### Plasma catecholamine concentrations

**Fig 6:**
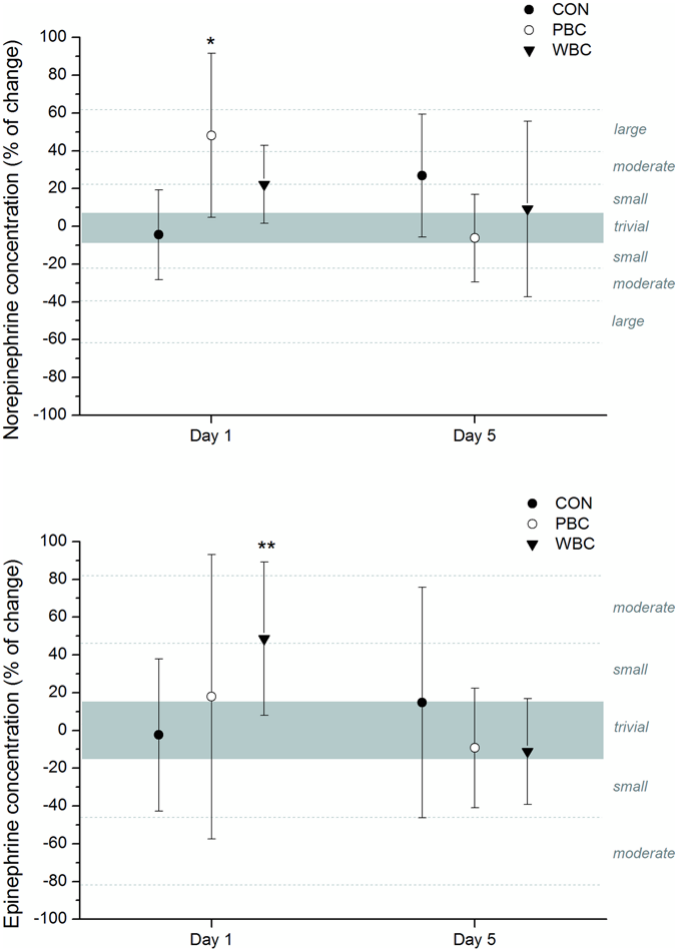
Changes (% of pre values) in plasma concentration in catecholamines from pre to post whole-body cryostimulation (WBC), and partial-body cryostimulation (PBC) sessions, and in the control (CON) condition. The shaded area represents trivial changes. Values were recorded on the first (1^st^ day) and fifth (fifth day) session. Between-group (PBC or WBC vs. CON) difference in the change: * likely; ** very likely; *** almost certain.

Cold-induced changes in plasma catecholamine concentrations are depicted in Fig 6. The first cryostimulation session *likely* induced a moderate to large increase in plasma norepinephrine concentration without any significant difference between PBC and WBC. These changes in plasma norepinephrine concentrations compared with baseline values were associated with a *likely* moderate increase in plasma epinephrine for WBC only. No further significant changes in plasma catecholamine concentrations were recorded for the last WBC and PBC sessions.

## Discussion

Numerous studies have accumulated scientific evidence supporting the beneficial effects of whole-body cryotherapy in the medical domain as an alternative treatment or rehabilitation technique, as well as in the sporting realm, especially in the context of post-exercise recovery. However the physiological mechanisms responsible for the effects of extreme cold exposure and the scientific basis for an optimal cryotherapy protocol remain unclear. In recently published work from our team, we showed that the stimulation of the autonomic nervous system after a single whole-body cryostimulation session was more pronounced by using a cryochamber system (exposing the whole body to a -110°C air) compared to an open tank (exposing the whole body except the head to a -160°C expanded nitrogen gas) [13]. As the cryochamber system was found to induce the largest decrease in skin temperature, leading to question whether the cold intensity or the head exposure to cold may be the key factor to induce physiological adaptations in cryotherapy. The next stage of research, presented in this article, was the study of the specific/isolated influence of head exposure to cold during five daily cryostimulation sessions inducing the same decrease in skin temperature.

### Effect of head exposure to cold on thermal responses

In order to study the effect of head exposure to cold, one of the big challenges of this experiment was to induce a similar decline in skin temperature of body regions exposed to cold except the head, in the two cryotherapy systems. After several preliminary testing sessions, it appeared that 3-min in a -60°C closed cryochamber (WBC) or in a -160°C open cryotherapy tank (PBC) induced an *almost certain* and similar very large reduction in Tskin of all body regions of interests (except the head). This result indicates that the PBC system had lower impact on skin temperature compared to the WBC system, mainly explained by the dissipation of nitrogen gas in the temperate environment by using the PBC open system. The mean decrease in Tskin for PBC was of 8.3°C (i.e. -26.1%) and 8.6°C (i.e. -26.9%) for WBC after the first cryostimulation session, and was *very likely* larger than after the fifth cryostimulation session (-7.6°C and -7.7°C for PBC and WBC, respectively). This study is one of the first to report the evolution of skin temperature after several consecutive cryostimulation sessions [34]. In both modalities, a *very likely* large reduction in Tskin of the head region was recorded after the sessions and in larger proportion after WBC (-7.6°C) than PBC (-1.5°C), which persisted up to 5min after the exposure. This large decrease in head temperature after WBC sessions was associated with a large decrease in Ttymp (-0.28°C) persisting up to 20min after the exposure. A small to moderate decrease in Ttymp was recorded after PBC sessions, only significant 20min after the exposure (-0.14°C), suggesting potentially lower physiological modifications when PBC is used as opposed to WBC, and without pronounced attenuation over the sessions (Fig 3). Similarly, Costello et al. [35] and recently Hausswirth et al. [13] reported significant reductions in Ttymp (-0.30 and -0.32°C, respectively) after a WBC session at -110°C. In the present study, cooling the head induced a larger core cooling as inferred through Ttymp values. Very few studies have tested the effects of whole-head cooling alone on whole-body thermal response, and mainly included partial exposure of the dorsal head in water. The main results indicate that dorsal head immersion in cold water alone (i.e. without body exposure to cold) has no effect on whole-body temperature, whereas when the body is exposed to cold water, additional submersion of the dorsal head dramatically increases core cooling by 39% in 12°C water to 87% in 10°C water [36,37]. In the first ethically approved study to evaluate the isolated contribution of whole-head cooling, Pretorius et al. [38] reported 12.6°C (62%) and 11.7°C (59%) decreases in Tskin for whole body with and without head submersion (after 30min in 17°C water), respectively. Similarly to our current findings, whole-head submersion induced a larger decline in core temperature (-0.84°C and -1.17°C in head-out and head-in conditions, respectively), even when the body was insulated in a 1.5mm thick vulcanized rubber dry suit covering the whole body except the head (-0.47°C and -0.77°C in head-out and head-in conditions, respectively). Data from our study seem to confirm that whatever the cold stimulus (water submersion or WBC), whole-head cooling might induce a larger thermal stimulus for peripheral vasoconstriction by increasing the body surface exposed to cold and by activating the trigeminal nerve afferents, reducing the perfused body mass and leading to a reduction of the thermal core [39–41]. Moreover, it is noteworthy that in our study, the top of the forehead and ears were protected from cold by wearing an earband during the cryostimulation sessions, possibly lowering the thermal stimulus applied on the head.

### Effect of head exposure to cold on the autonomic nervous system

The question of the optimal reduction in skin or core temperature is still a matter of debate. According to the recent study of Hausswirth et al. [13], it seems that a larger reduction in body temperature could induce a greater stimulation of the autonomic nervous system, as inferred though blood catecholamine concentrations and HRV indices. However, whether cooling the head in addition to the rest of the body contributes to these effects is not known and constitutes the main question of this article. In the present study, plasma norepinephrine concentration was *likely* increased after the first cryostimulation session in similar proportions between cryotherapy modalities. Thus, cooling the head in the WBC modality had no more effect on the adrenergic response than PBC. Further, the increase in plasma norepinephrine (+22.2% after the 1^st^ WBC session) was lower than that classically reported in studies using lower cold temperatures in cryochambers (-110°C), suggesting that the magnitude of the adrenergic response may be mainly related to the cold intensity. In comparison, Hausswirth et al. [13] reported a 76.2% increase in norepinephrine concentration after one 3-min WBC session at -110°C. Additionally, in the present study, no marked increase in norepinephrine concentration was recorded after the fifth WBC and PBC sessions, suggesting a lower sensitivity to the thermal stimulus. Moreover, it is noteworthy that the catecholamine half life is short and because of logistical consideration in the analysis of skin temperature, HRV and BP, blood draws were done ~25min after the exposition to cold, period of time during which the catecholamine concentration probably came back down.

During cryostimulation, cold-sensitive cutaneous receptors excite afferent neurons, which in turn stimulate sympathetic nerve activity. Norepinephrine release stimulates α-adrenergic receptors in the vasculature, resulting in peripheral vasoconstriction. Consequently, blood flow is redistributed toward the core, resulting in increased arterial blood pressure, as previously reported to occur after WBC sessions [13,42]. In the present study, Dia BP was *likely* increased in small proportion in response to the moderate increase in norepinephrine, confirming a lower sympathetic stimulation with a smaller cold stimulus. Moreover the increase in Dia BP was only observed after the two first cryostimulation sessions and only in the WBC modality, confirming an attenuation of the sympathetic response with consecutive cryostimulation sessions, and showing that despite its small surface area the head may contribute to the global cold-related vasoconstriction mechanism. The decrease in Ttymp recorded only after WBC sessions, as well as the stimulation of cold trigemino-cardiac reflex receptors located in the face may have accentuated the parasympathetic response after WBC, augmenting vagal output to the heart. Despite small effects of cryostimulation sessions on BP, HR was largely decreased after WBC (mean decrease over the week: -12.3%) and PBC (-8.6%) sessions, without significant difference between the first and the last session, suggesting that the cold-induced bradycardia would not be mainly a baroreflex mediated mechanism in cryotherapy. Khuruna et Wu [18] also reported that the bradycardia induced by the head submersion in water occurred before the increase in BP, and this cardiodeceleration effect may be probably larger by using cool water. According to these results, the cold-related bradycardia might be mainly mediated by an increase in activity of the parasympathetic branch of the autonomic nervous system, likely thanks to the faster conduction velocity of parasympathetic fibers compared to sympathetic fibers [43]. Further, the larger but not significant bradycardia recorded after WBC than PBC could be explained by the additional stimulation of trigeminal cold receptors located in the face and the greater body surface exposed to cold (+7–10%) [38].

HRV indices of parasympathetic activity (RMSSD and HF) were *likely* to *very likely* increased after each cryostimulation session without differences between WBC (RMSSD: +38.8%; HF: + 70.3%) and PBC (RMSSD: +49.1%; HF: +122.3%) over the week. The increases in HRV values recorded in this study for WBC and PBC are lower than that reported in previous studies using -110°C cryochambers. For example, Schaal et al. [15] reported increases of 78% and 296% in RMSSD and HF, respectively after a single WBC session at -110°C in elite synchronized swimmers, while Hausswirth et al. [13] reported increases of 54% and 138% of RMSSD and HF, respectively after a single PBC session at -160°C in recreationally active men. Taken together, data from the present study and the literature suggest that the magnitude of parasympathetic response to cryostimulation would be related to the magnitude of the cold stimulus rather than to the head exposure to cold. Further, like for HR response the increases in RMSSD and HF parameters were the same from the first to the last cryostimulation session whatever the cryotherapy system. Contrarily to the sympathetic response, which was attenuated with subsequent cryostimulation sessions, the parasympathetic response was maintained up to the last session. Since WBC (at -60°C) and PBC (at -160°C) are able to repeatedly induce a moderate but consistent increase in parasympathetic activity, these techniques should be recommended specifically as a chronic treatment to stimulate the parasympathetic tone regularly, for example when physical exercise bouts are repeated such as during training camps or multistage competitions.

### Implications for the use of different techniques of cryotherapy

This study has practical implications regarding the use of cryotherapy:-it confirms the interest of using cryotherapy when parasympathetic stimulation is sought;—it raises new insight on the use of head-in or head-out cryotherapy systems;—it shows the effects of acute vs. chronic cryotherapy treatments.

The results of this study support the use of cryotherapy, particularly when parasympathetic stimulation is sought. Elevated parasympathetic activity at rest is classically associated with health and well-being, and is enhanced by regular physical activity and more generally by a healthy lifestyle, including balanced diet, no smoking and no alcohol [44]. Body exposure to cold is an effective method to increase parasympathetic activity easily and rapidly and greater effects are obtained by using air-based cryotherapy protocols [13,15,16] than cold water immersion [9,10,45].

Two main modalities of air-based cryotherapy are currently employed (i.e. head exposed or not exposed to cold), prompting questions on the usefulness of each of these techniques and on physiological mechanisms involved. In the present study, both cryotherapy techniques induced a quite similar parasympathetic stimulation without marked effect of head cooling on recorded variables. These results suggest that the intensity of the autonomic response (i.e. parasympathetic stimulation) would be mainly proportional to the cold intensity, rather than the head cooling. It can be supposed also that the short-term cold exposure (3-min) in cryotherapy may not be sufficient to trigger a parasympathetic stimulation through the trigeminal nerve endings, trigeminal afferents being mostly unmyelinated fibers suggesting latency in their response [46]. Finally, wearing a protective earband and surgical mask during cryostimulation sessions may probably lower the sensibility of trigeminal receptors to cold. Within this context, the use of colder temperatures (i.e. -110°C, generally reported in the literature) would be recommended in order to enhance the parasympathetic stimulation, likely by stimulating a greater number of cold receptors.

Finally knowing whether the physiological responses to cold attenuate along with repeated cryostimulations sessions is of great interest for practitioners to propose adapted prescriptions to athletes and patients. Although blood catecholamine concentration was altered only after the first cryostimulation session, changes in HR, BP and HRV persisted in similar proportions up to the last session. These results may be related to a decrease in the sympathetic response with time, whereas the parasympathetic response would be maintained. From a practical point of view, this finding suggests that WBC at -60°C or PBC at -160°C could be preferentially used as chronic treatments when a regular stimulation of parasympathetic activity is needed, for example in rehabilitation purposes. On the other hand, when a rapid and large parasympathetic stimulation is needed such as in post exercise recovery, the use of a colder temperature of WBC would be more appropriate as an acute treatment. However, whatever the cryotherapy treatment (acute vs. chronic and head-in or head-out), to date we do not know how long lasts the cryostimulation-induced increase in parasympathetic activity. This knowledge could be determinant for the prescription of cryotherapy in sport recovery and to enhance sleep quality.

## Conclusion

The present study confirms that a WBC or PBC session induces an immediate stimulation of the ANS, as inferred from heart rate variability indices and blood catecholamine concentrations. A predominance of the parasympathetic tone was recorded from the first to the fifth WBC and PBC sessions, with small differences between groups in the magnitude of the response. Both cryotherapy modalities induced the same acute reduction in Tskin and quite similar physiological reactions from the first to the fifth cryostimulation session. Only Ttymp and HR were slightly more reduced after WBC than PBC, traducing a trend to a greater parasympathetic response after WBC than PBC. According to these results, cooling or not cooling the head during cryostimulation sessions may have a small influence on the modulation of the ANS. However, taken together with other studies, these data suggest that the key factor influencing the body response to cryostimulation might be the magnitude of the reduction of body temperature rather than the head cooling. Additional studies are warranted in order to identify the optimal level of body cooling in cryotherapy and how long parasympathetic activity can be increased after a cryostimulation.
